# Manipulating Cellular Factors to Combat Viruses: A Case Study From the Plant Eukaryotic Translation Initiation Factors eIF4

**DOI:** 10.3389/fmicb.2019.00017

**Published:** 2019-02-05

**Authors:** Corinne Schmitt-Keichinger

**Affiliations:** UMR_A 1131 Santé de la Vigne et Qualité du Vin, INRA – Université de Strasbourg, Colmar, France

**Keywords:** virus resistance, loss-of-susceptibility, recessive, engineered resistance, eIF4E, CRISPR/Cas9, hairpin, host factor

## Abstract

Genes conferring resistance to plant viruses fall in two categories; the dominant genes that mostly code for proteins with a nucleotide binding site and leucine rich repeats (NBS-LRR), and that directly or indirectly, recognize viral avirulence factors (Avr), and the recessive genes. The latter provide a so-called recessive resistance. They represent roughly half of the known resistance genes and are alleles of genes that play an important role in the virus life cycle. Conversely, all cellular genes critical for the viral infection virtually represent recessive resistance genes. Based on the well-documented case of recessive resistance mediated by eukaryotic translation initiation factors of the 4E/4G family, this review is intended to summarize the possible approaches to control viruses via their host interactors. Classically, resistant crops have been developed through introgression of natural variants of the susceptibility factor from compatible relatives or by random mutagenesis and screening. Transgenic methods have also been applied to engineer improved crops by overexpressing the translation factor either in its natural form or after directed mutagenesis. More recently, innovative approaches like silencing or genome editing have proven their great potential in model and crop plants. The advantages and limits of these different strategies are discussed. This example illustrates the need to identify and characterize more host factors involved in virus multiplication and to assess their application potential in the control of viral diseases.

## Introduction

Plants have evolved sophisticated processes to evade pathogen attacks. The main antiviral defense responses are considered to be RNA silencing, *R* gene mediated resistance and recessively inherited resistance, although other cellular mechanisms like autophagy (Hafren et al., 2017; Haxim et al., 2017; Li et al., 2018), RNA methylation (Martínez-Pérez et al., 2017) or ubiquitination (Alcaide-Loridan and Jupin, 2012) are also important to counter viruses.

RNA silencing constitutes a widespread defense mechanism against viruses harboring RNA or DNA genomes. dsRNA fragments, either formed during viral replication or as secondary structures in viral transcripts, are processed by DICER-like RNases (DCLs) into short interfering RNAs (siRNAs). The so-called guide strand then takes the RNA-induced silencing complex (RISC), containing argonaute (AGO) proteins, to complementary RNAs which leads to their degradation or translational inhibition. This defense response is counteracted via different mechanisms by viral proteins known as viral suppressors of RNA silencing (for reviews on antiviral silencing and VSR mechanisms, see Garcia-Ruiz et al., 2016; Zhao et al., 2016).

In the gene-for-gene resistance, a plant encoded *R* gene product recognizes a viral factor called avirulence factor (Avr), either in a direct interaction or indirectly *via* modifications caused by the Avr on plant factors, according to the guard or decoy models (Dangl and Jones, 2001; Moffett et al., 2009). R proteins are mainly of the NLR family (or NBS-LRR as a reference to their nucleotide binding site and leucine-rich repeat domain). They interact with viral effector proteins displaying various functions in the virus life cycle. This interaction leads to an incompatible reaction where a hypersensitive response (HR) takes place. HR is characterized by programmed cell death and generally results in virus containment at its site of entry. This resistance response referred to as effector triggered immunity is genetically conditioned by both the plant and the pathogen and any allelic variation that impairs this recognition changes the response to a compatible reaction.

Besides these dominant *R* genes many resistance genes with recessive inheritance have been described and some of these have been characterized. From a conceptual point of view, a recessive resistance either reflects a mutation in a plant factor essential for the virus life cycle or a mutation in a regulator of plant defense (Truniger et al., 2009; Hashimoto et al., 2016). The first kind of mutation (in a dominant gene) renders the pathogen unable to highjack important cellular functions and is therefore termed loss-of-susceptibility or non-host resistance; it is considered passive. The second kind of mutation leads to the absence or dysfunction of a negative regulator of plant defense responses resulting in a continuous autoactivation of these responses; it thus represents an active resistance. The latter has long remained theoretical for virus resistance, until the report in 2013 (Orjuela et al., 2013) that the rice *RYMV2* resistance could be caused by a loss-of-function mutation in a gene similar to the *Arabidopsis thaliana* regulator of defense *constitutive expresser of pathogenesis-related genes-5* (CRP5, Love et al., 2007; Fujisaki et al., 2009).

Loss-of-susceptibility can be acquired by mutation(s) in virtually any gene implied in a cellular mechanism needed at any step of the viral infection cycle including translation, replication or movement. Indeed, replication of tobamoviruses is impaired in *ARL8* or *TOM1* mutants (Nishikiori et al., 2011) whereas replication of red clover necrotic mosaic dianthovirus needs Hsp proteins (Mine et al., 2012). The movement of two viruses of the family *Geminiviridae* was found impaired in the Pla1 accession of *A. thaliana* (Reyes et al., 2017) and long distance spread deficiency accounts for the *bc-1* resistance to a potyvirus in common bean (Feng et al., 2017). However, the vast majority of recessive resistance has been linked to translation. This is the case for the well-known initiation factors eIF4E, eIF4G and their isoforms (collectively referred to as eIF4 factors) but also for the guanine nucleotide exchange factor eIF2Bß conferring resistance to the turnip mosaic potyvirus (Shopan et al., 2017). The EXA1 protein conferring resistance to potexviruses potentially regulates translation *via* its eIF4E binding motif (Hashimoto et al., 2016) and the *Pelo* gene controlling begomovirus resistance is involved in ribosome recycling (Lapidot et al., 2015).

It is anticipated that more plant factors involved in virus amplification will be discovered and their mechanism elucidated in the coming years. This represents a promising resource for genetic resistance against viruses. Taking the eIF4 factors as an example, this review presents some possible approaches to turn our knowledge on virus-host interactions into antiviral strategies. From classical breeding to genome editing, all kinds of methods have been applied to model and crop plants to exploit eIF4-based resistance.

## eIF4-Factors in Plants

**FIGURE 1:**
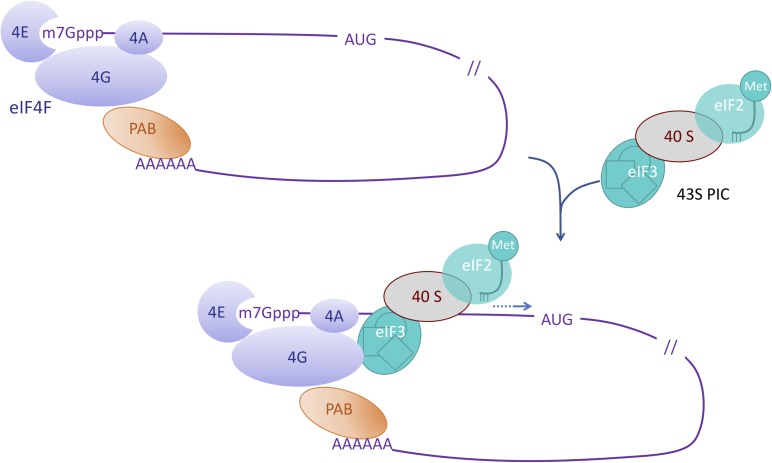
Role of eIF4 factors in the initiation of mRNA translation. This simplified scheme depicts the eIF4F complex composed of eIF4E (4E) which interacts with the cap (m7Gppp) of the mRNA, eIF4G (4G) which interacts with both eIF4E and the polyA bound PolyA binding protein (PAB), and eIF4A (4A). Interactions between eIF4G and eIF3 enable the recruitment of the pre-initiation complex (PIC) containing the initiator Met-tRNA and the 40S ribosomal subunit. During virus infection interactions like the one between eIF4 factors and the viral VPg are suggested to highjack the translation machinery.

eIF4 factors are critical proteins involved in the initial step of the translation of eukaryotic mRNAs. eIF4E factors bind to the cap (a 7-methylated guanosine linked to the 5′ end of the transcript through a 5′-5′ triphosphate bond, noted m7Gppp) while a polyadenosine binding protein PABP binds the 3′ poly(A) tail of the transcript (Figure 1). eIF4G factors are large scaffold proteins that can bind eIF4E factor, PABP and RNA thus circularizing the mRNA (For a review on eukaryotic mRNA translation initiation, see Merrick and Pavitt, 2018). eIF4G together with eIF4E and the DEAD (Asp-Glu-Ala-Asp) containing helicase eIF4A form the eIF4F complex. The eIF3 factor is part of the 43S preinitiation complex (PIC) also containing the 40S ribosomal subunit and the eIF2-GTP-Met-tRNA ternary complex (TC). Interactions between eIF3 in the PIC and eIF4G allow for the recruitment and stabilization of the PIC at the vicinity of the cap structure. The PIC complex then moves along the mRNA until it reaches the initiation codon, which is generally the first AUG in a favorable context. This represents the scanning step that precedes the reorganization of the initiation complex, triggering the recruitment of the large ribosomal subunit and the beginning of the elongation step.

In plants, three members of the eIF4E protein family have been described (Hernández and Vazquez-Pianzola, 2005): eIF4E and eIF(iso)4E both belonging to class I and the novel cap binding protein nCBP, now renamed 4EHP and belonging to class II (Dinkova et al., 2016). Higher plants also possess at least two eIF4G isoforms: eIF4G and eIF(iso)4G. Interactions preferentially occur between eIF4E and eIF4G to form eIF4F and between eIF(iso)4E and eIF4(iso)4G to form eIF4(iso)4F. 4EHP was described to interact with eIF(iso)4G but displayed a poor efficiency in promoting *in vitro* translation (Dinkova et al., 2016). Although providing some selectivity, these isoforms also ensure some redundancy, as suggested by the absence of strong phenotypes in eIF4E or eIF(iso)4E knock out mutants (Yoshii et al., 1998; Duprat et al., 2002; Sato et al., 2005; Bastet et al., 2017).

Besides the translation of cap-bearing cellular or viral RNAs, translation factors are also involved in the non-canonical translation of viral RNAs devoid of a cap structure. Various elements of the translational initiation complex have been described to interact with viral RNAs (Sanfaçon, 2015 and references therein). Such an interaction has been widely found between the genome linked protein VPg of potyvirids, for example, and eIF4 factors (Léonard et al., 2000; Schaad et al., 2000; Robaglia and Caranta, 2006).

## eIF4-Based Recessive Resistance

In the 1990s, recessively inherited resistance sources were identified against viruses from the family *Potyviridae* (Provvidenti and Hampton, 1992). These recessive genes were either from cultivars or from closely related plants and represented about 40% of the known genes conferring resistance to potyviruses. Based on the knowledge acquired from different models that the genome linked potyviral encoded protein VPg interacted with the eIF4E factor, that resistance-breaking determinants mapped to the VPg-coding sequence and that the VPg-eIF4E interaction was crucial for virus multiplication, a gene candidate approach was used to identify the pepper *eIF4E* gene as the first natural recessive gene conferring resistance to potato virus Y (PVY), tobacco etch virus (TEV, Ruffel et al., 2002) and lettuce mosaic virus (LMV, Duprat et al., 2002). The same year, a map-based cloning of *Arabidopsis* mutants identified *eIF(iso)4E* as the resistance gene to turnip mosaic virus (TuMV, Lellis et al., 2002).

Since then many other eIF4 factors were cloned or shown to co-segregate with recessive resistance to viruses (Robaglia and Caranta, 2006; Sanfaçon, 2015). These factors are predominantly eIF4E variants conferring resistance to potyviruses through the loss of the eIF4E-VPg interaction. However, some viruses or virus strains preferentially use eIF(iso)4E, eIF4G or eIF(iso)4G factors for their life cycle. Thus, mutations or variations in these isoforms were also reported to confer resistance to a variety of viruses, some with smaller VPg, like poleroviruses (Reinbold et al., 2013) and some with no VPg at all, like the turnip crinkle carmovirus and the cucumber mosaic cucumovirus (Robaglia and Caranta, 2006; Sanfaçon, 2015). Also, translation does not seem to be the only step of the virus cycle in which the eIF4 factors are involved. Hence cell-to-cell movement (Gao et al., 2004) and systemic spread (Contreras-Paredes et al., 2013) of two potyviruses were proposed to rely on eIF4E and eIF(iso)4E factors, respectively. Recently, the cell-to-cell spread of the plantago asiatica mosaic potexvirus was shown to be delayed in plants mutated for the 4EHP (nCBP) factor, due to the impaired accumulation of two movement proteins translated from subgenomic viral RNAs (Keima et al., 2017).

Although the mechanisms of the recessive resistance conferred by eIF4 factors are not all understood, it has been widely tested mostly because it can target a large variety of viruses in a wide range of plants from both the monocotyledon and dicotyledon clades, in model or crop plants (barley, rice, pea, tomato…).

## Engineering eIF4-Based Resistance

### Classical Breeding

**FIGURE 2:**
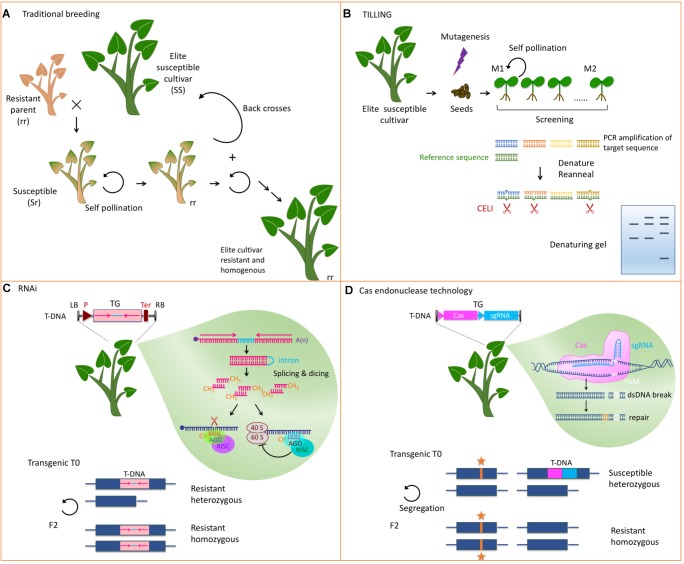
Strategies to develop host factors involved in virus multiplication into resistance. **(A)** Classical breeding to introgress a recessive resistance gene from a relative into an agronomically well performing susceptible cultivar. Many rounds of selfings, backcrosses, and selections are needed to get a homogenous resistant cultivar. **(B)** TILLING allows screening mutants in a candidate gene. DNA is extracted from mutagenized seedlings and the candidate DNA fragment is PCR amplified. A similar fragment from a reference plant is allowed to hybridize and mismatches between the reference DNA and the mutant DNA are recognized by an endonuclease like CELI. Fragments are analyzed on a denaturing polyacrylamide/agarose gel and compared to a wild type control. **(C)** RNAi uses a short hairpin RNA obtained from the transcription of a transgene made of two inverted repeats from the target sequence (pink) separated by an intron (light blue). The silencing machinery of the plant dices this dsRNA and represses the expression of the cognate endogenous gene through slicing of the mRNA (blue) or inhibition of its translation. This strategy leads to a dominant resistance. TG, transgene; LB and RB, left and right border; P, promoter of transcription; T, terminator of transcription. **(D)** Genome editing techniques allow precise modifications of the genome. A Cas endonuclease, and a single guide RNA (sgRNA) are expressed from a transgene. They induce dsDNA breaks at the site targeted by the sgRNA next to the protospacer adjacent motif (PAM). The cellular machinery then repairs the DNA either by non-homologous end joining or by homologous DNA repair if a template DNA is present. Self-pollination of the transgenic mutated plants allows segregation of the mutation (noted by an orange star) and the transgene. Subsequent generations are thus devoid of transgenic sequences. Lower panels in **(C,D)** depict the modified loci and illustrate the homozygous or heterozygous status needed for resistance.

Due to their recessive inheritability, loss-of-susceptibility genes are considered challenging in breeding programs but they are expected to provide durable and broad spectrum resistance like non-host resistance (Pavan et al., 2010). Introgression of such genes requires a series of backcrosses with the elite parent followed by self-crosses and by the selection of the offspring for resistance and agronomic traits (Figure 2). Breeding schemes are therefore long and laborious and some undesirable traits may be impossible to eliminate. Testing of resistance to viruses include symptomatology and ELISA and can be assisted by molecular markers as exemplified for pea resistance to pea seed borne mosaic virus (PSbMV, Smýkal et al., 2010). Traditional breeding of loss-of-susceptibility genes presents the advantage of being well accepted by the society but requires the identification of resistance genes in plants genetically compatible with the cultivar of interest. Such natural variants of eIF4 factors have been identified in several plants including pepper (*Capsicum annuum*) lettuce (*Lactuca sativa*), pea (*Pisum sativum*) or barley (*Hordeum vulgare*) and have been introgressed in many cultivars (Ruffel et al., 2002; Nicaise et al., 2003; Gao et al., 2004; Stein et al., 2005).

### Tilling and EcoTilling

In tobacco plants (*Nicotiana tabacum*) X-ray mediated mutagenesis was applied to generate the *va* recessive resistance (Koelle, 1958) which has since been identified as an eIF4 encoding gene (Julio et al., 2015). In the intervening years this resistance gene was introgressed into all tobacco cultivars commercially available (Korbecka-Glinka et al., 2017). This example illustrates the possibility to engineer resistance through random mutagenesis and selection. Based on the known potential of variant alleles of eIF4 to confer resistance, mutagenesis techniques were coupled with an effective screening method to generate and select mutations in these particular genes in a reverse genetics approach. TILLING (Targeted Induced Local Lesions IN Genomes) takes advantage of ethyl methanesulfonate (EMS) that leads to saturated chemical mutagenesis and high-throughput screening techniques to detect polymorphism in a targeted sequence (Kurowska et al., 2011). After DNA isolation and PCR amplification of the fragment of interest, heteroduplexes are identified mainly using single stranded specific endonucleases like the celery (*Apium graveolens*) deriving CELI (Figure 2). Alternatively, more specific mutagenic agents or next generation sequencing can be employed. EcoTILLING is defined as a method using the mutation detection technologies of TILLING to find polymorphism in natural populations (Comai et al., 2004).

TILLING and EcoTILLING can be used when no resistance gene is known, to generate or identify allelic variants of any important host factor. It can create or detect different types of mutations including point mutations like missense changes or stops, truncation or mutations in splice junction sequences (Kurowska et al., 2011). As TILLING does not require transformation it is applicable to recalcitrant or non-transformable species and resulting resistant plants are exempted from European GMO regulations. Also, TILLING is not technically challenging and is relatively cost-effective.

Such a TILLING approach was successfully used to produce a tomato splicing mutant of eIF4E1 that exhibited immunity toward a PVY strain and a TEV strain, which correlated with a loss of cap binding activity (Piron et al., 2010). In *Cucumis* spp, EcoTILLING was applied to 113 accessions to identify an eIF4 allelic variant controlling resistance against melon necrotic spot virus (MNSV). The amino-acid substitution lies outside of the cap binding pocket but could still regulate the cap binding activity of eIF4E (Nieto et al., 2007).

Experiments in tomato highlighted the difficulty of generating loss-of-function mutations without affecting resistance spectrum or plant growth even in the case of gene redundancy like for eIF4 factors (Gauffier et al., 2016). In this tomato/potyvirus pathosystem a knock out engineered *eIF4E1* mutant appeared to present a narrower resistance spectrum than the natural resistance allele that encodes several amino acid substitutions within the eIF4E1 protein, probably because eIF4E1 regulates the availability of eIF4E2 for the virus (Gauffier et al., 2016). TILLING offers the advantage of generating a series of allelic variants among which functional variants mimicking natural resistance genes can be preferred to loss-of-function mutants.

### Silencing, Overexpression, and Synthetic Gene Engineering

Knocking-down susceptibility factors to trigger virus resistance can also be obtained through silencing in transgenic plants (Figure 2). Expression of short intron-spliced hairpin-containing RNAs (hpRNAs) with homology to the desired eIF4 target efficiently provided resistance in numerous hosts including tomato (Mazier et al., 2011), cucumber (Rodríguez-Hernández et al., 2012) and plum trees (Wang et al., 2013). The self-complementary sequence of the hairpin can be chosen according to the needed selectivity toward different eIF4 isoforms. In cucumber, a hairpin designed to silence *eIF4E* but not *eIF(iso)4E* triggered resistance to three viruses in the family *Potyviridae* and one in the family *Tombusviridae* (Rodríguez-Hernández et al., 2012). Silencing the tomato *eIF4E1* and *eIF4E2* without affecting *eIF(iso)4E* led to large anti-potyviral resistance (Mazier et al., 2011), whereas in plum, specific silencing of *eIF(iso)4E* but not *eIF4E* provided effective resistance to plum pox virus (PPV) the causal agent of Sharka (Wang et al., 2013).

RNAi has the advantage of being effective on polyploid hosts as shown by the recent work on the allotetraploid tobacco plant (Takakura et al., 2018). By silencing both homeologous genes *eIF(iso)4E-S* and *eIF(iso)4E-T* (respectively, inherited from the *N. sylvestris* or *N. tomentosiformis* parent) the authors could decrease the accumulation of a resistance breaking strain of PVY. They combined this approach with the screening of an EMS mutant library to further demonstrate the implication of the eIF(iso)4E-T but not the eIF(iso)4E-S isoform in the virulence of this PVY strain escaping the resistance provided by the deletion of *eIF4E1-S*.

Less intuitively, overexpression of a host factor involved in the multiplication of a plant virus can also provide genetic resistance to this virus. Either a natural resistant variant or a non-functional mutant of the susceptibility factor is overexpressed in transgenic plants in order to outcompete the interaction between the virus and the endogenous functional factor. This dominant negative approach has been successfully described in several studies and represents a good way to transfer virus resistance in crops that have not developed eIF4-based resistance. Mutations in the overexpressed gene either mimic existing variants, derive from protein-protein interaction data or combine these two options (Kang et al., 2007; Yeam et al., 2007; Cavatorta et al., 2011; Kim et al., 2014). The two transgenic strategies were also mixed to overexpress an eIF4E1 variant in potato plants silenced for the native eIF4E1 factor (Duan et al., 2012).

More recently, transgenesis was used to complement an *Arabidopsis* line knocked out for *eIF4E1* with a synthetic *eIF4E* gene combining 6 amino-acid substitutions existing in natural pea alleles. The broad resistance obtained in these *Arabidopsis* plants illustrates the great potential of using gene engineering to exploit natural variability (Bastet et al., 2018).

Transgenesis or intragenesis (if the gene from the target crop is modified) transforms the recessivity of the resistance to a dominantly inherited trait. It is also relatively easily applicable to various cultivars and thus preserves existing crop diversity. In the absence of public acceptance for final marketable crops this technology still represents a valuable and efficient tool to study the relevance of given gene modifications that can then be obtained by other means.

### Targeted Genome Modifications

Techniques to precisely target sequences to be modified in plant genomes have recently been developed, based on DNA repair after double strand breaks provoked by sequence-specific endonucleases like zinc-finger nucleases (ZFNs), transcription activator-like effectors nucleases (TALENs), and CRISPR (clustered, regularly interspaced, short palindromic repeat) associated (Cas) endonucleases. These technologies often compared to molecular scissors allowing targeted mutagenesis or short indels are continuously improving and their use to generate antiviral resistance is rapidly increasing (Cardi and Stewart, 2016; Komor et al., 2016; Zaidi et al., 2016; Murovec et al., 2017; Romay and Bragard, 2017). The efficacy of Cas technology to generate eIF4-based resistance was first reported in cucumber (Chandrasekaran et al., 2016) and *Arabidopsis* (Pyott et al., 2016) and more recently in cassava (Gomez et al., 2018) where mutations were respectively introduced into eIF4E, eIF(iso)4E and nCBP. In these three studies mutations caused small indels leading to the knock-out of the targeted isoform and to resistance toward viruses of the family *Potyviridae*.

However, knock-out alleles of redundant genes present the risk of limited efficiency in virus resistance because of their possible impact on the expression of other isoforms or because viruses can adapt to recruit other factors (Gauffier et al., 2016; Bastet et al., 2017). Therefore, non-synonymous mutations seem to be preferable to knock-out alleles. This kind of point mutations are achievable with the Cas editing tools, either by inducing homology-directed DNA repair through the presence of a template DNA fragment harboring the desired sequence (donor repair DNA) or by using modified Cas proteins harboring nucleotide modification activities instead of the endonuclease activity (Cardi and Stewart, 2016; Komor et al., 2016; Begemann et al., 2017; Lu and Zhu, 2017). These improvements in the precision and multiplexing of mutations open the way to non-transgenic plants with synthetic functional alleles showing broad and durable resistance to viruses.

Gene editing represents a powerful tool to improve crops and seems to be only limited by the delivery of the entire expression system (Cas, guide RNA and potentially the donor repair molecule) and the efficacy in regenerating plants from cell or tissue culture. It has the advantage of being versatile and producing plants that do not contain foreign genes. The engineered plants are similar to mutated cultivars or natural variants that have been used for long time in traditional breeding programs. No doubt that the recent decision by the US Department of Agriculture not to regulate plants that have been modified through genome editing will prompt viral resistance into a new era. On the contrary, the European Court of Justice has recently ruled that edited plants fall within the scope of the GMO directive and do not even benefit from the exemption granted to plants mutated by use of older techniques with a “long safety record.” This view of considering living beings according to the techniques used for their generation is not without practical and ethical problems.

## Concluding Comments

eIF4-based resistance to viruses perfectly illustrates the recent progress in engineering loss-of-susceptibility resistance, from the observation of a recessive resistance to the latest rational synthetic gene design. These experiences could help in defining a general approach applicable to other host factors. In a first step, high throughput techniques such as transcriptomics, yeast-two-hybrid or virus-induced gene silencing could identify new host partners of viral factors. The second step would consist in evaluating the biological importance of the interaction for the virus life cycle and evaluate the possibility to manipulate the host gene, that is assess the specificity of the interaction in case of a multigene family and evaluate the possibility to manipulate that gene without affecting important agricultural traits. A crucial development resides in obtaining or identifying alleles disrupting the interaction with the virus factor without greatly affecting the plant. Screening methods like reverse yeast two hybrid (White, 1996) or EcoTILLING could assist this task and the resulting candidates could be reproduced via genome editing in the desired crop varieties.

An evaluation of crop performance, of resistance spectrum and of possible interference with other viruses will then need large-scale field trials. The durability of resistance is an important parameter and mathematical models as well as experimental studies are aimed at assessing and improving it (Kobayashi et al., 2014). Combining multiple resistance genes and resistance strategies should help advance sustainable disease control (Quenouille et al., 2013; Kobayashi et al., 2014; Fuchs, 2017). All the recently developed high throughput technologies associated with genome editing techniques will undoubtedly help favoring agriculture in the everlasting arms race between plants and viruses.

## Author Contributions

CS-K wrote the manuscript and drew the figures.

## Conflict of Interest Statement

The author declares that the research was conducted in the absence of any commercial or financial relationships that could be construed as a potential conflict of interest.
